# Tobacco on the Eyes

**Published:** 1888-12

**Authors:** 


Tobacco on the Eyes.—The eyes of some are especially susceptible to tobacco. Those who feel the slightest nausea or giddiness after using tobacco should let it alone.

There is a woman in this city who smoked only one cigar a day for three years, but who at the end of that time was afflicted with tobacco amaurosis of both eyes; nor has she ever regained her sight.—*Keyser.*

DANGER IN LARGE DOSES OF QUININE.—Quinine sometimes affects the eyes dangerously. He relates the case of a man who came to him from Ohio, several years ago, totally blind.

This man had, in the attempt to doctor himself for an attack of malaria, taken two doses of quinine, an hour apart, each consisting of a heaping tablespoonful of the drug. Blindness followed in a few hours; and treatment proved of no avail; the man's sight is still wanting.—*Keyser.*

SIX CHILDREN AT A SINGLE BIRTH.—At Dallas, Texas, on November 3d, Mrs. Geo. Hirsh, of Navarro County, gave birth to six children. The mother and children are doing well. There are four boys and two girls. All are perfect and fully proportioned, but very small. The babies are all tagged, to preserve their identity.

